# Therapeutic potential of five frequently prescribed herbs in obesity-associated Hashimoto’s thyroiditis: insights from efferocytosis regulation

**DOI:** 10.3389/fmed.2025.1538867

**Published:** 2025-05-19

**Authors:** Jiahao Zhou, Tianshu Gao

**Affiliations:** ^1^The First Clinical College, Liaoning University of Traditional Chinese Medicine, Shenyang, China; ^2^Department of Endocrinology, Affiliated Hospital of Liaoning University of Traditional Chinese Medicine, Shenyang, China

**Keywords:** hashimoto’s thyroiditis, obesity, quercetin, efferocytosis, network pharmacology, molecular docking

## Abstract

**Background:**

Patients with obesity-associated Hashimoto’s thyroiditis (HT) have been prevalent in clinical practice. Obesity is a risk factor for HT as it promotes pro-inflammatory processes and influences the balance of immune cell subsets. Traditional Chinese medicine (TCM) is characterized by its multi-component and multi-target approach and shows potential in treating HT. Specifically, TCM can reduce thyroid antibody levels and alleviate clinical symptoms without impairing thyroid function. Moreover, TCM offers significant benefits in regulating lipid metabolism and decreasing systemic inflammation.

**Methods:**

Targets of five high-frequency herbs (*Hedysarum multijugum* Maxim, Radix Bupleuri, *Prunella vulgaris*, *Fritillaria thunbergii* Bulbus, and *Angelicae sinensis* Radix) were obtained from the TCMSP and Swiss Target Prediction databases. Targets associated with obesity-associated HT were collected from the GeneCards, OMIM, and DisGeNET databases. Subsequently, we employed KEGG signaling pathway enrichment and GO biological process enrichment analyses to investigate the potential mechanisms by which the active ingredients of these herbs treat obesity-associated HT. Then, STRING database networks and Cytoscape software were used to construct the protein-protein interaction network and screen for key targets. Finally, molecular docking was performed to predict the binding interactions between the targets.

**Results:**

Efferocytosis emerged as the key mechanism in the context of five herbs and obesity-associated HT. Quercetin was identified as the primary active ingredient responsible for efferocytosis, and it bound well with efferocytosis-related targets.

**Conclusion:**

This study’s key finding is that five high-frequency prescribed herbs may treat obesity-associated HT through efferocytosis. This provides new evidence to support the use of TCM in treating obesity-associated HT.

## 1 Introduction

Hashimoto’s thyroiditis (HT), also known as chronic lymphocytic thyroiditis, is one of the common autoimmune diseases (ADs) in clinical treatment. Modern medicine believes that the etiology of HT is associated with genes, environment, and epigenetic factors (1). Patients with HT and normal thyroid function are mainly treated by nutritional therapy and lifestyle intervention (2–5). If patients suffer hypothyroidism or subclinical hypothyroidism, doctors usually apply levothyroxine alternative therapy (6). TCM has the potential to reduce thyroid antibody levels and improve clinical symptoms without affecting thyroid function and has been evidenced in clinical efficacy and safety (7, 8). To date, antioxidant, immune regulation, and anti-inflammatory properties are the main mechanisms of active ingredients of herbs for HT (6, 9–13).

Obesity serves as a risk factor for HT (14). Clinical studies have shown that it can impact thyroid function and antibody levels in patients with HT (15, 16). In addition, obesity promotes pro-inflammatory processes and influences the balance of immune cell subsets (14). Research on both obesity and HT has largely focused on the perspective of inflammation and immune dysfunction (14, 17).

Efferocytosis is a process in which macrophages specifically recognize and engulf apoptotic cells (18). The process of efferocytosis is divided into three steps: recognition of dying cells, engulfment of dying cells, and digestion of dying cells (18–21). At the same time, efferocytosis can also produce anti-inflammatory factors (20). Recent studies demonstrated that atherosclerosis, aging, cancer, obesity, diabetes, and some ADs are related to efferocytosis (20, 22–25). However, HT has not been mentioned. There were also targeted clinical trials for efferocytosis targets (AXL, MERTK, CD47, Tim4, and Tim3) (20).

Quercetin is a flavonoid compound in many fruits and vegetables (26). Studies have found that quercetin has the effect of regulating immunity (27), promoting apoptosis (28), and upregulating PPAR-*γ* signaling (29). In addition, quercetin inhibits macrophage M1 polarization and promotes M2 polarization (30), and M2 macrophages are associated with efferocytosis (31).

The objective of this study was to explore the potential mechanisms of five herbs in treating obesity-associated HT by using network pharmacology and molecular docking.

## 2 Materials and methods

### 2.1 Collection of herb and disease targets

The targets of *Hedysarum multijugum* Maxim (HMM), Radix Bupleuri (RB), *Prunella vulgaris* (PV), *Fritillaria thunbergii* Bulbus (FTB), and *Angelicae Sinensis* Radix (ASR) were identified by searching the Traditional Chinese Medicine Systems Pharmacology (TCMSP) database, PubChem database, and Swiss Target Prediction database. Obesity-related targets were searched from the GeneCards database, OMIM database, and DisGeNET database using “Obesity” as the search term. The GeneCards and OMIM databases were used to search for targets using “Hashimoto thyroiditis” as the search term. Finally, three obesity disease datasets and two HT disease datasets were merged to obtain the obesity-associated HT disease genes.

### 2.2 GO and KEGG pathway enrichment analyses

KEGG pathway enrichment and GO biological process enrichment (including biological processes, molecular functions, and cellular components) were performed on five single herbs and four herb pairs (PV-HMM, PV-FTB, PV-RB, and HMM-ASR) by the Medscape database (*p* < 0.05). Venn diagrams were employed through the Venn mapping website to analyze the similarities and differences in mechanisms of the enrichment analysis (32).

### 2.3 Analysis of efferocytosis-related active ingredients

We matched targets of the herb’s active ingredient and herb-related efferocytosis to obtain the most active component. To further verify that the active ingredients are related to efferocytosis, the intersection targets of the compound and disease were matched using the Venn mapping website and then imported into the Metascape database.

### 2.4 Protein–protein interaction network construction and target protein screening

A total of 38 efferocytosis targets obtained from five herbs were imported into the STRING database to obtain the PPI network of efferocytosis. CytoHubba was used to screen the top 15 key targets. Cytoscape software was used to construct the target network.

### 2.5 Molecular docking

Molecular docking was performed between the active ingredient and key targets. AXL and MERTK were also included as key targets because they are present in 38 efferocytosis targets and have been clinically validated. The potential proteins were entered into the PDB database to obtain the associated protein structure, downloaded in PDB format, and saved. The AutoDock Vina software was used for molecular docking. The targets were treated by removing water molecules and performing hydrogenation. Receptor was then selected and then exported as a PDBQT file. Quercetin was treated by hydrogenation and selected as the ligand. It was then exported as a PDBQT file. Finally, the interaction strength was obtained by molecular docking of the target protein and quercetin. A molecular docking process is deemed valid when the calculated binding free energy is less than 5.0 kcal/mol, indicating a stable interaction between the ligand and the receptor.

## 3 Results

### 3.1 Intersections between five high-frequency herb targets and obesity-associated HT targets

A total of 531 HMM targets, 234 FTB targets, 449 RB targets, 334 PV targets, and 94 ASR targets were predicted. A total of 2,296 HT targets were retrieved, with 1,832 from GeneCards and 537 from OMIM. A total of 5,671 obesity targets were acquired, with 5,271 targets from GeneCards, 521 targets from OMIM, and 300 targets from DisGeNET. The targets of obesity and HT were merged, resulting in 6,711 obesity-associated HT targets (Supplementary Table S1).

### 3.2 The GO enrichment results of five high-frequency herbs and four herb pairs

**Figure 1:**
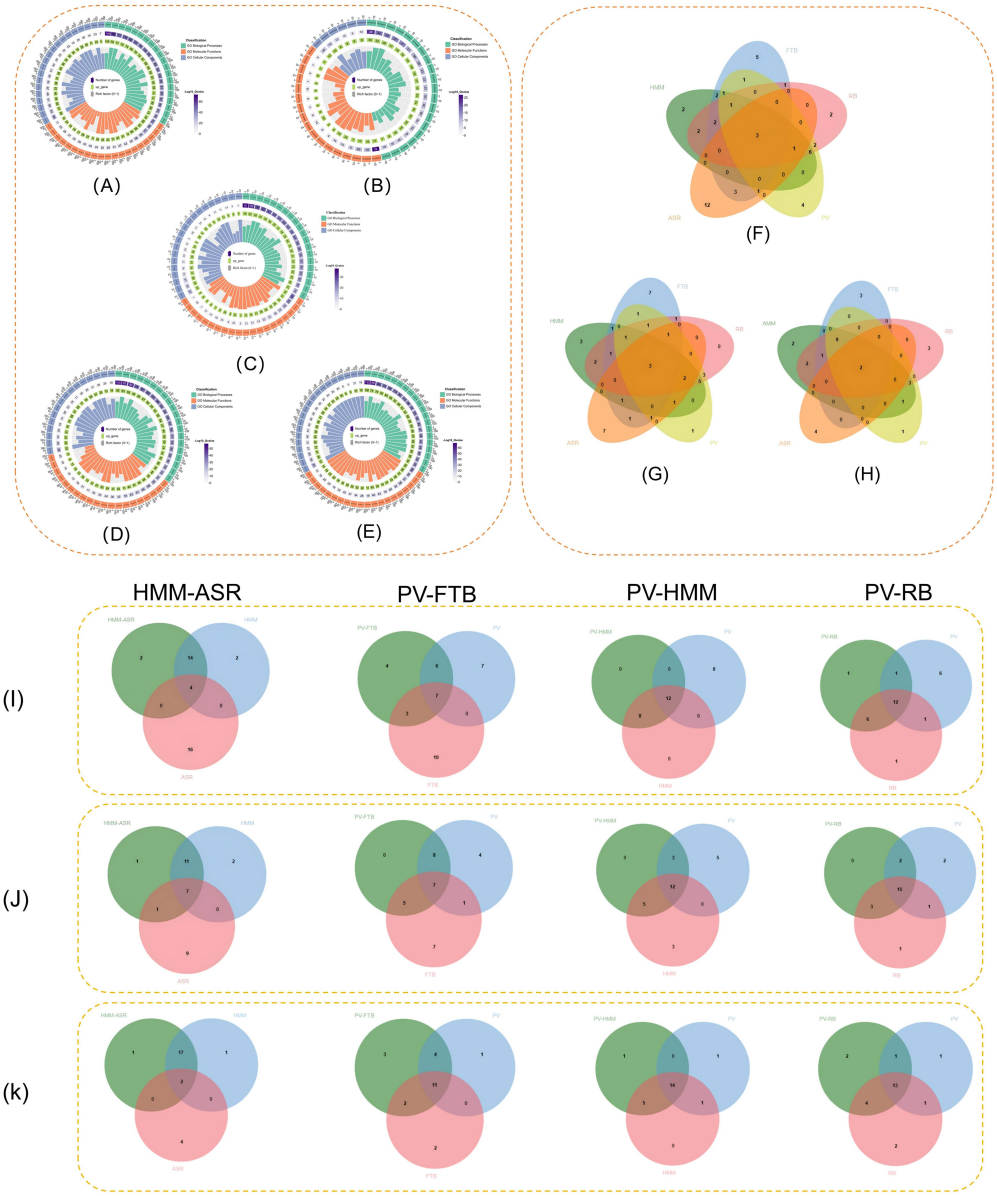
**(A)** GO enrichment analysis of HMM. **(B)** GO enrichment analysis of ASR. **(C)** GO enrichment analysis of FTB. **(D)** GO enrichment analysis of PV. **(E)** GO enrichment analysis of RB. **(F)** Venn diagram of five-herb BP analysis. **(G)** Venn diagram of five-herb MF analysis. **(H)** Venn diagram of five-herb CC analysis. **(I)** Venn diagram of four-herb pair BP analysis. **(J)** Venn diagram of four-herb pair MF analysis. **(K)** Venn diagram of four-herb pair CC analysis. Bubble figure of five high-frequency herbs and four herb pair KEGG enrichment analysis.

Through GO analysis, we identified that all five herbs were enriched in response to hormone (GO: 0009725). HMM, RB, PV, and ASR were enriched in the positive regulation of programmed cell death (GO: 0043068) (Figure 1F; Table 1; Supplementary Table S2). Furthermore, HMM, RB, and PV were enriched in six identical biological processes, one of which was the response to oxidative stress (GO: 0006979) (Figure 1F; Supplementary Table S2). The MF results revealed that all five herbs were enriched in transcription factor binding (GO: 0008134), protein domain-specific binding (GO: 0019904), and neurotransmitter receptor activity (GO: 0003707). HMM, RB, PV, and ASR were enriched in oxidoreductase activity (GO: 0016491) (Figure 1G; Table 1; Supplementary Table S2). The cellular component (CC) analysis indicated that these five herbs showed significant enrichment in GO terms related to the receptor complex (GO: 0043235) and organelle outer membrane (GO: 0031968) (Figure 1H; Table 1; Supplementary Table S2). In the biological process (BP) enrichment analysis, HMM and RB were both enriched in response to oxidative stress (GO: 0006979), with a GeneRatio value of 0.91 (Figures 1A,E; Supplementary Table S3). The entry for the positive regulation of programmed cell death (GO: 0043068) was also significantly enriched in PV, with a GeneRatio value of 0.84 (Figure 1D; Supplementary Table S3). In the MF enrichment analysis of the five herbs, HMM, RB, PV, and ASR were all enriched in oxidoreductase activity (GO: 0016491), with a GeneRatio value of 1 (Figures 1A,B,D,E; Supplementary Table S3). The Venn diagram results of the BP enrichment analysis showed that the four herb pairs and their related individual herbs were all enriched in response to hormone (GO: 0009725) (Figure 1I; Supplementary Table S3). In addition, the PV-RB and PV-HMM pairs intersected with their related single herb in response to oxidative stress (GO: 0006979) (Figure 1J; Supplementary Table S3). MF enrichment analysis showed that the HMM-ASR pairs intersected with their related herbs on oxidoreductase activity (GO: 0016491) and hormone binding (GO: 0042562) (Figure 1J; Supplementary Table S3).

**Table 1:** Venn diagram interaction between five high-frequency herbs.

	BP	MF	CC
Five herbs	Response to hormone	Transcription factor binding	Receptor complex
	Response to xenobiotic stimulus	Protein domain-specific binding	Organelle outer membrane
	Regulation of body fluid levels	Neurotransmitter receptor activity	
HMM, RB, PV, and ASR	Positive regulation of programmed cell death	Oxidoreductase activity	
HMM, RB, PV, and FTB		Phosphatase binding	
HMM, RB, and PV	Response to oxidative stress		

### 3.3 The KEGG enrichment analysis of five high-frequency herbs and four herb pairs

**Figure 2:**
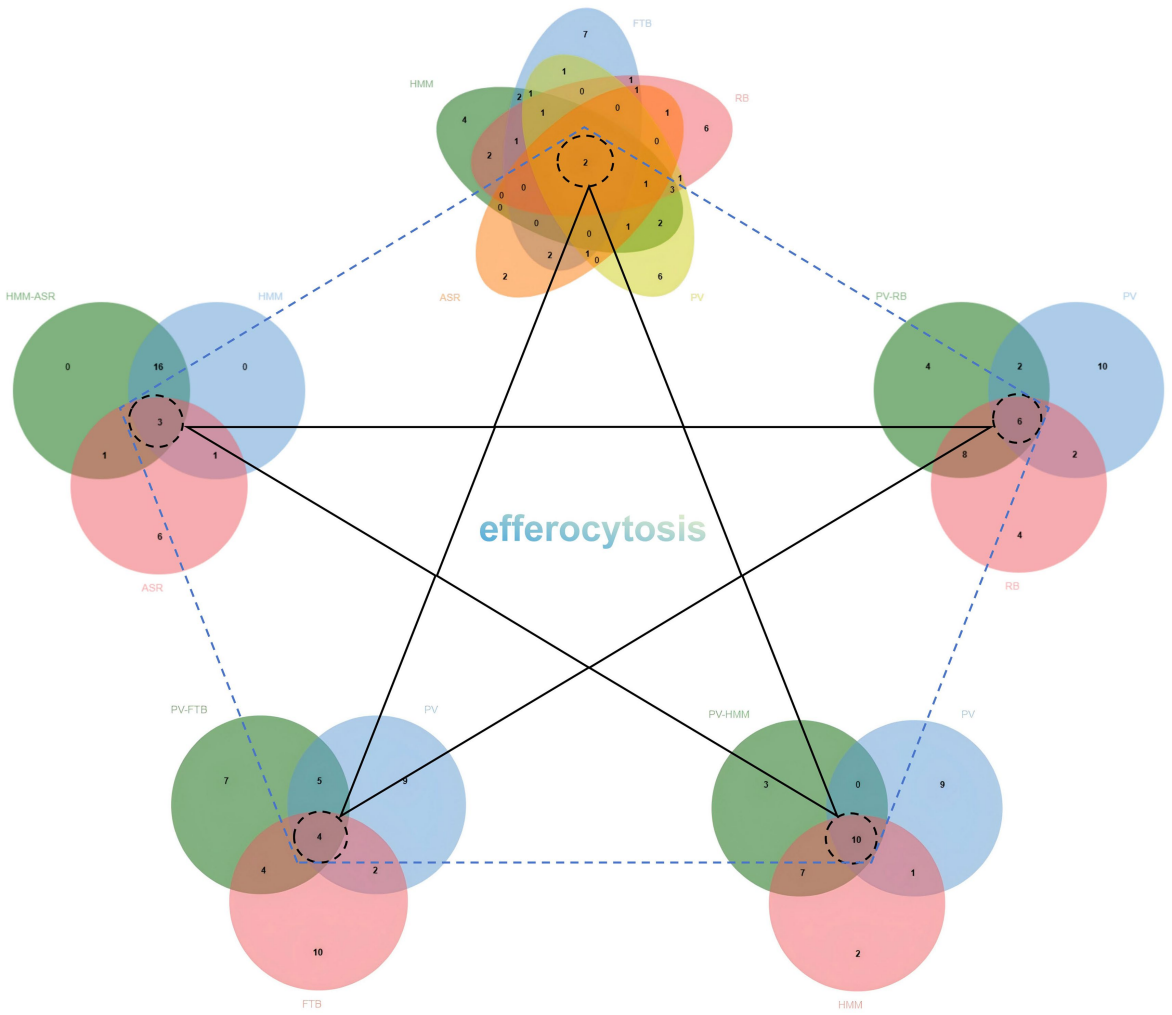
KEGG enrichment intersections between five herbs and four herb pairs.

**Figure 3:**
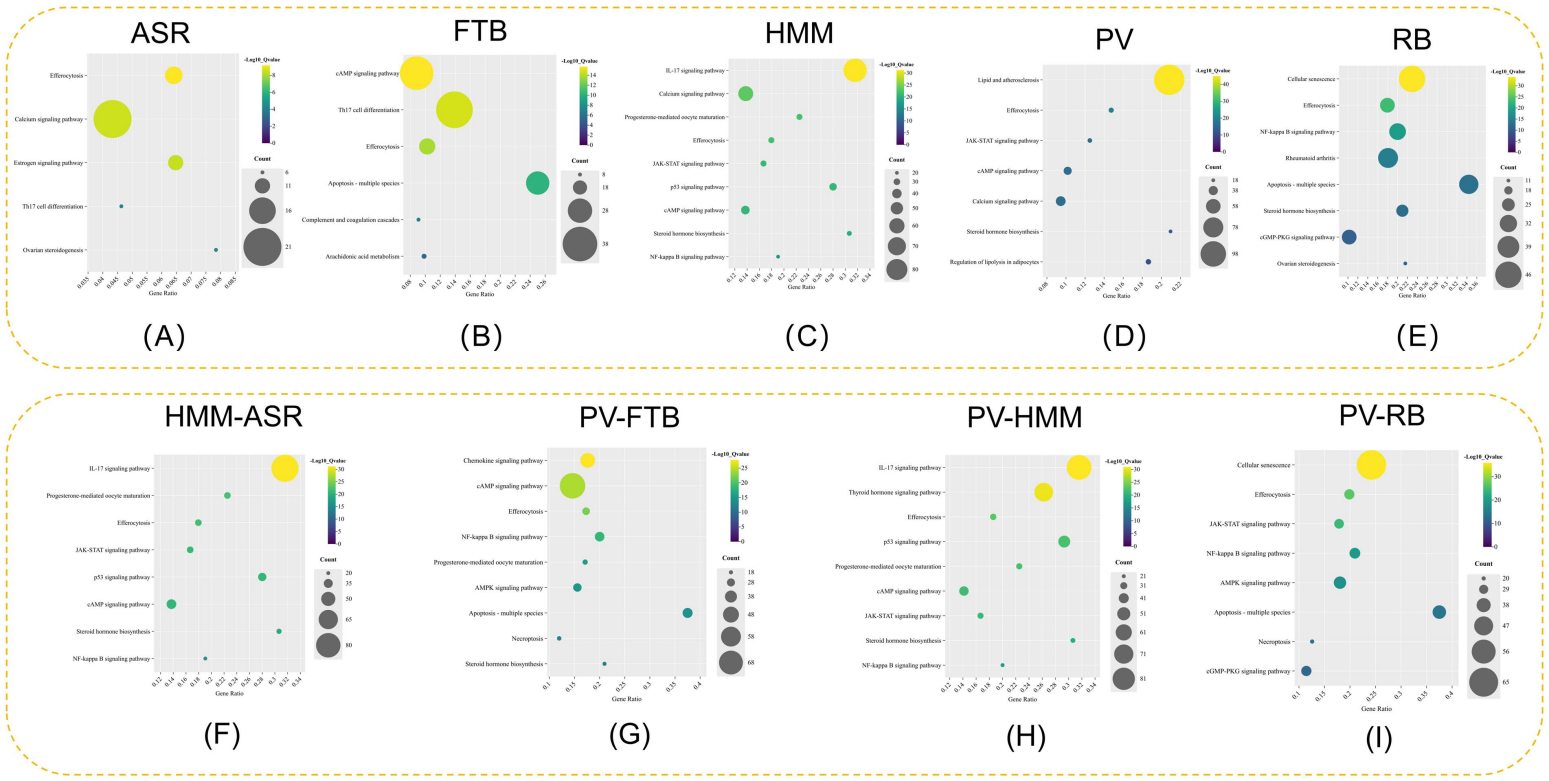
**(A)** KEGG enrichment analysis of ASR. **(B)** KEGG enrichment analysis of FTB. **(C)** KEGG enrichment analysis of HMM. **(D)** KEGG enrichment analysis of PV. **(E)** KEGG enrichment analysis of RB. **(F)** KEGG enrichment analysis of HMM-ASR. **(G)** KEGG enrichment analysis of PV-FTB. **(H)** KEGG enrichment analysis of PV-HMM. **(I)** KEGG enrichment analysis of PV-RB.

KEGG enrichment analysis results showed that all of the herbs were enriched in the pathways of cancer (hsa04270) and efferocytosis (hsa04148) (Figure 2). In addition to efferocytosis, pathways related to inflammation, immunity, hormones, and lipid metabolism were screened for bubble figures. IL-17 signaling pathway (hsa04657) was the most significantly enriched item in KEGG of HMM, with a GeneRatio value of 0.32 (Figure 3C; Table 2). PV was enriched in lipid and atherosclerosis (hsa05417), with a GeneRatio value of 0.208 (Figure 3D; Table 2). The first two enriched pathways of FTB were Th17 cell differentiation (hsa04659) and apoptosis—multiple species (hsa04215), with GeneRatio values of 0.14 and 0.25 (Figure 3B; Table 2). The first item enriched in RB was cellular senescence (hsa04218), with a GeneRatio value of 0.23 (Figure 3E; Table 2). ASR was enriched in the estrogen signaling pathway (hsa04915) (Figure 3A; Supplementary Table S4). The significance of gene ratio is the ratio of active ingredients and pathway intersection genes to total pathway genes. Efferocytosis pathways for HMM, RB, and PV were among the top 20 significantly enriched pathways, and the GeneRatio values of HMM and RB were both 0.18 (Table 2). Venn diagrams and bubble figures indicated that the four herb pairs and five herbs were all significantly enriched in efferocytosis (hsa04148) (Figure 2). In addition, the thyroid hormone signaling pathway (hsa04919) is unique to the PV-HMM herb pair compared to the PV and HMM (Figure 3H; Supplementary Table S4). The AMPK signaling pathway (hsa04152) appeared only in PV-RB and PV-FTB herb pair (Figures 3G,I; Supplementary Table S4). The GO enrichment results of FTB and the CC analysis results of the four drug pairs did not show significant rules (Figures 1C,K).

**Table 2:** Top 20 KEGG enrichment analysis terms for HMM, FTB, RB, PV, and ASR.

Herb	ID	GeneRatio	Description
RB	hsa04215	0.34375	Apoptosis—multiple species
HMM	hsa04657	0.31578947	IL-17 signaling pathway
HMM	hsa00140	0.30645161	Steroid hormone biosynthesis
HMM	hsa04115	0.28	p53 signaling pathway
FTB	hsa04215	0.25	Apoptosis—multiple species
RB	hsa04218	0.22929936	Cellular senescence
HMM	hsa04914	0.22522523	Progesterone-mediated oocyte maturation
RB	hsa04913	0.21568627	Ovarian steroidogenesis
RB	hsa00140	0.20967742	Steroid hormone biosynthesis
PV	hsa00140	0.20967742	Steroid hormone biosynthesis
PV	hsa05417	0.20833333	Lipid and atherosclerosis
RB	hsa04064	0.2	NF-kappa B signaling pathway
HMM	hsa04064	0.19047619	NF-kappa B signaling pathway
PV	hsa04923	0.18644068	Regulation of lipolysis in adipocytes
RB	hsa05323	0.18085106	Rheumatoid arthritis
HMM	hsa04148	0.17948718	Efferocytosis
RB	hsa04148	0.17948718	Efferocytosis
HMM	hsa04630	0.16666667	JAK–STAT signaling pathway
PV	hsa04148	0.1474359	Efferocytosis
FTB	hsa04659	0.13888889	Th17 cell differentiation

### 3.4 KEGG enrichment analysis of quercetin in efferocytosis

**Figure 4:**
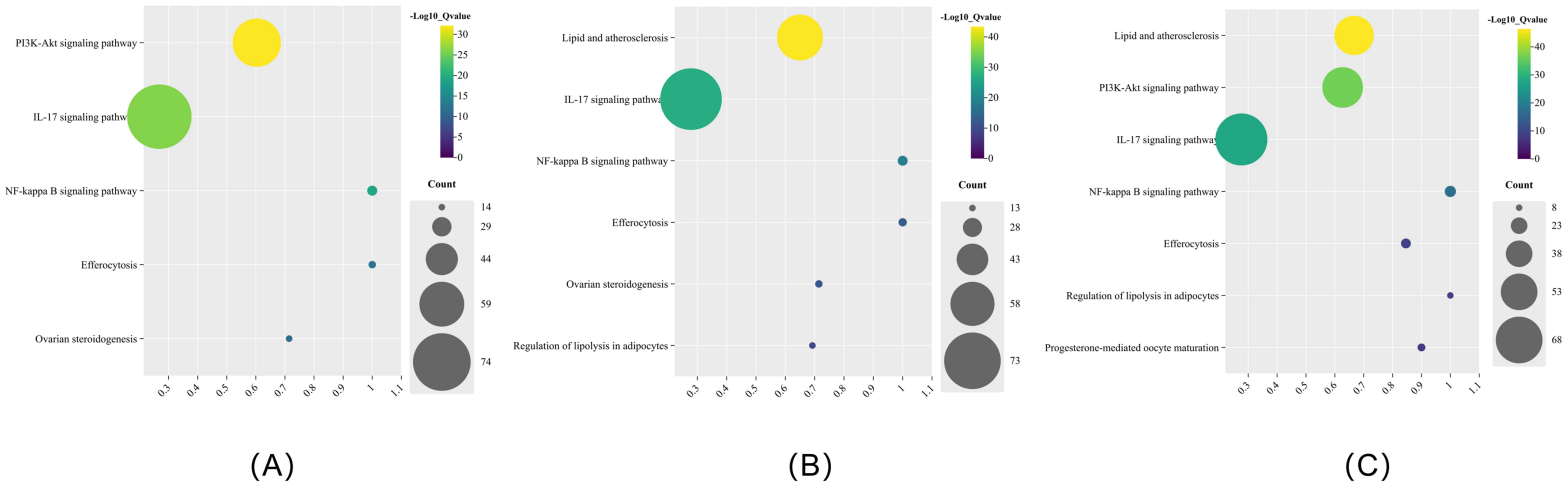
Bubble figure of quercetin KEGG enrichment analysis. **(A)** Bubble figure of quercetin and obesity-HT. **(B)** Bubble figure of quercetin and obesity. **(C)** Bubble figure of quercetin and HT.

Quercetin was found to be one of the active ingredients by matching the targets of herbs and efferocytosis (Supplementary Table S5). The enrichment results indicated that efferocytosis was one of the pathways for quercetin and obesity-associated HT (Figure 4A). We further performed KEGG enrichment of the intersection of quercetin-HT targets and quercetin-obesity targets (Figures 4B,C). As shown in Figure 4, we observed that in addition to efferocytosis, the IL-17 signaling pathway and NF-κB signaling pathway were also the interaction pathways for the three conditions.

### 3.5 Protein–protein interaction network construction and screening of key targets

**Figure 5:**
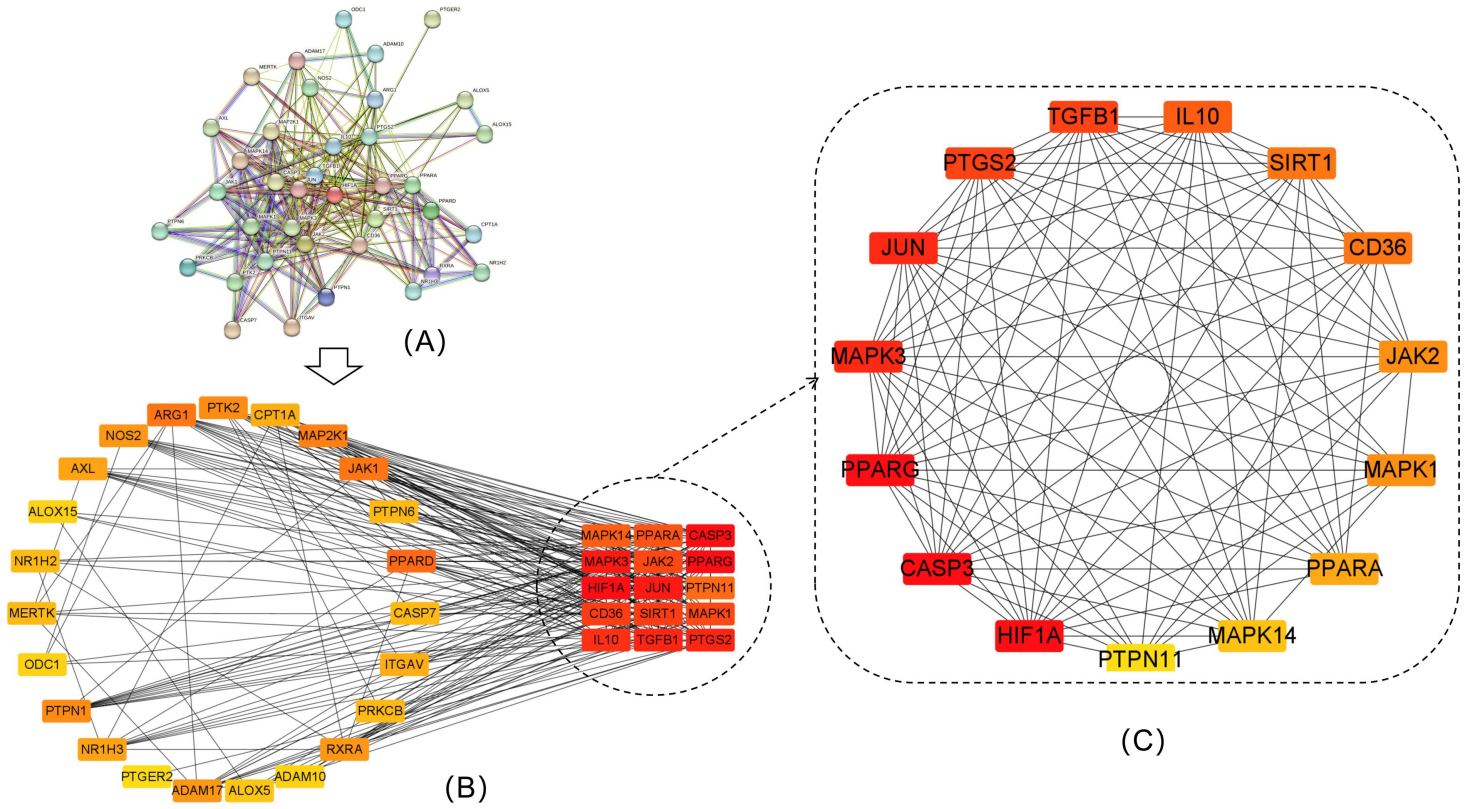
PPI network of intersecting targets and top 15 intersecting targets. **(A)** PPI network of the 38 efferocytosis targets. **(B)** Visualization of 38 efferocytosis targets by Cytoscape software. **(C)** The top 15 core targets for efferocytosis.

A total of 38 proteins and 267 edges were obtained after the 38 efferocytosis targets were imported into the STRING database (Figure 5A), and then, the data were imported into Cytoscape to obtain the PPI network diagram (Figure 5B). The top 15 key nodes screened were CASP3, PPARG, HIF1A, MAPK3, JUN, PTGS2, TGFB1, IL10, CD36, SIRT1, JAK2, MAPK1, PPARA, MAPK14, and PTPN11 (Figure 5C). Reducing PPARG signaling results in defective efferocytosis (20). Other targets do not have direct evidence of relevance to efferocytosis, and even if they bind well with quercetin, further experiments are needed to verify their relationship with efferocytosis.

### 3.6 Molecular docking results

The docking results showed that 17 targets were well bound to quercetin. MERTK and AXL; the results showed that all proteins bound well with quercetin. The docking scores of the 17 targets are shown in Table 3. Lower docking values indicate stronger binding affinities, with values less than −5.0 kcal/mol indicating potential binding and values less than −7.0 kcal/mol indicating strong binding affinities (33). MERTK, AXL, and PPARG are involved in the process of efferocytosis (20). Other proteins have not been reported to be directly involved in efferocytosis and require further experimental evidence.

**Table 3:** Binding energy of efferocytosis-related proteins.

Proteins	PDBID	Binding energy
CASP3	5IBP	7.2
PPARG	9F7W	7.6
HIF1A	2ILM	8
MAPK3	4QTB	7.7
JUN	6Y3V	6.4
PTGS2	5F19	8.7
TGFB1	4KV5	7.4
IL10	1INR	6.6
CD36	5LGD	8.5
SIRT1	8ANB	9.2
JAK2	3UGC	7.9
MAPK1	5WP1	7.5
PPARA	6KAX	8.2
MAPK14	3LFF	8.3
PTPN11	3ZM0	7.4
MERTK	7AB0	6.5
AXL	4RA0	7.4

## 4 Discussion

KEGG enrichment analysis showed that efferocytosis is the key pathway in obesity-associated HT. We also found that quercetin is the potential ingredient of efferocytosis. However, Cesidio et al. found that quercetin can inhibit the growth and function of normal thyroid cells (34). This toxic result is derived from *in vitro* cell experiments and animal experiments. In addition, although it has been reported that quercetin has an inhibitory effect on thyroid peroxidase, this conclusion was drawn from *in vitro* experiments on pig thyroids (35). It is well known that quercetin has a low oral bioavailability (36). It has also been mentioned that at flavonoid concentrations above 20 μM, the effects observed *in vitro* are hardly applicable to human intake of flavonoids through diet or dietary supplements (37). Plasma concentrations of flavonoids can reach 0.7 to 2.5 μM in subjects who consume large amounts of vegetables, such as onions, or who take dietary supplements (37). The toxic dose of quercetin based on animal experiments was equivalent to approximately 8 mg/kg in the human body (37). Therefore, whether it can reach toxic levels in the human body at normal doses of TCM applications requires further *in vivo* experiments to confirm. A relevant study has indicated that a daily intake of 1 g of quercetin, when used as a dietary supplement, is considered safe (38). Another study reported that the recommended dose of quercetin was 100–1,200 mg/d, with an intake of up to 2 g/d (37, 39). In addition, a clinical study mentioned that supplementation with quercetin at 500 mg/day and 1,000 mg/day for 12 weeks significantly increased plasma quercetin levels but did not affect innate immune function or inflammatory markers in adult women in the community (40). Therefore, compared with its toxicity, the ability of anti-inflammatory and regulate immunity should be more concerned (36).

Recent studies have shown that quercetin can regulate obesity and obesity-related complications (41–43). Quercetin has anti-inflammatory and anti-oxidative effects (44, 45); it also has been shown to have anti-obesity effects in adipocyte cultures and animal models (46). Jazyra Zynat et al. discovered that quercetin could significantly influence adipose tissue in obese conditions. It achieves this by mitigating intracellular oxidative stress, diminishing chronic low-grade inflammation, and suppressing adipogenesis and lipogenesis (44, 47). Mie Nishimura et al. have shown that quercetin-rich onion powder can reduce HDL cholesterol in obese people (48). In addition, it has been reported that quercetin can prevent obesity by regulating intestinal flora (49–51). It has also been reported that quercetin can intervene in obesity by regulating the expression of liver lipid metabolism-related genes and pro-inflammatory genes (50, 52).

Therefore, it is suggested that quercetin may have a potential intervention effect on efferocytosis. Quercetin has been experimentally demonstrated to treat obesity, and enrichment analysis showed that the efferocytosis was significantly enriched in the intersection genes of quercetin associated with HT. However, the intervention of quercetin on HT still needs further clinical studies.

The regulatory effects of quercetin on some ADs have been demonstrated by *in vivo* animal experiments, such as experimental osteoarthritis (53). The five herbs mentioned in this study are also frequently found in clinical compound experiments (6, 8), such as Shugan Sanjie Prescription (8), which includes five herbs. Buzhong Yiqi decoction is a traditional formula that contains RB, HMM, and ASR. In a study of the rat model of experimental autoimmune thyroiditis, Buzhong Yiqi granules significantly reduced antibody levels and improved thyroid function in the model group (54). A clinical study has also claimed that BuzhongYiqi decoction can enhance the immunity of the elderly (55). In addition, clinical studies have shown that PV can reduce thyroid antibody levels, improve thyroid function, and reduce thyroid volume (6, 56). HMM can reduce the levels of TPOAb and TgAb and promote the apoptosis of HT mice (6). Saikosaponin D, the active component of RB, could treat HT by regulating macrophage polarization (12).

**Figure 6:**
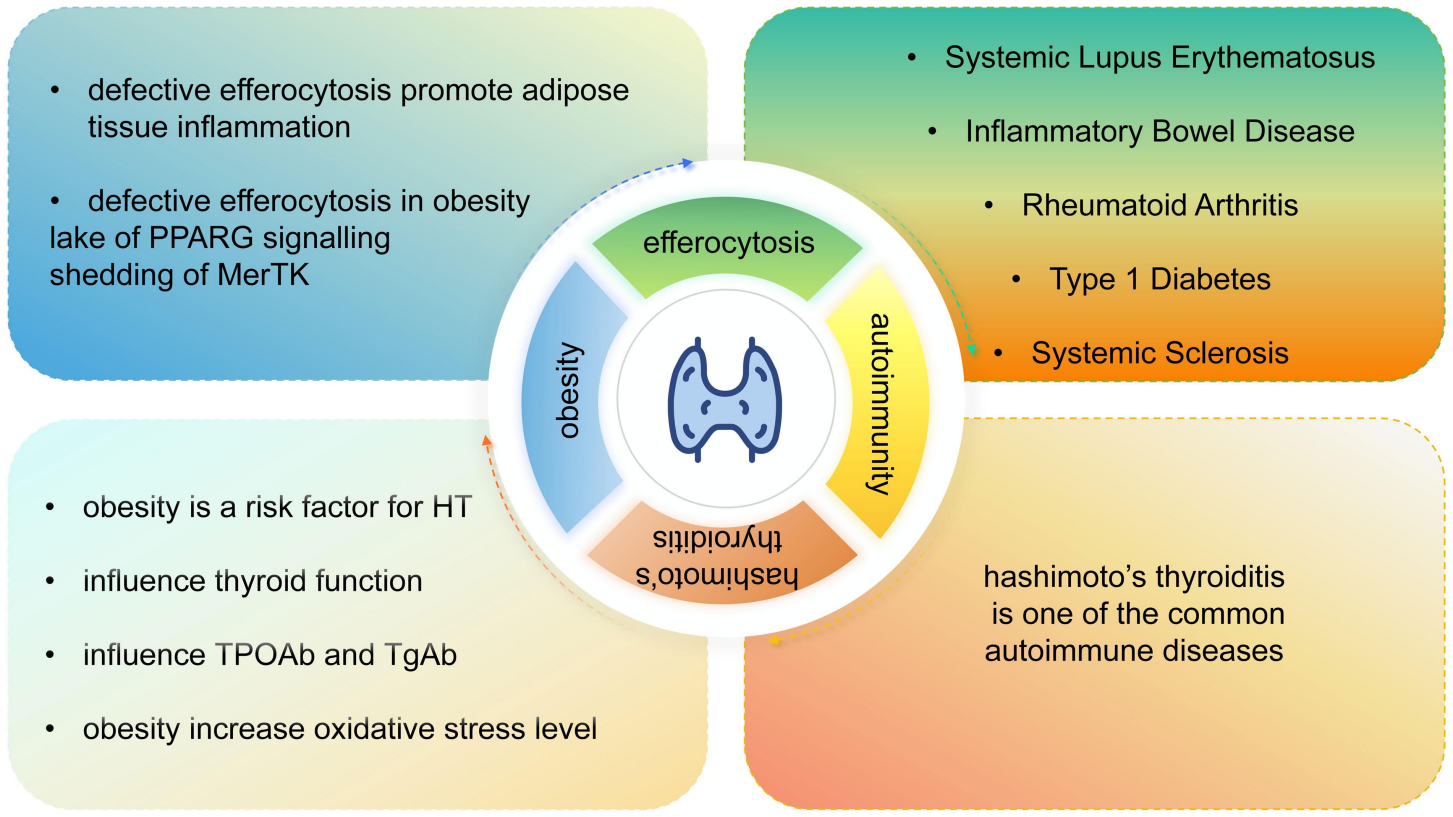
Relationship between HT, obesity, autoimmunity, and efferocytosis.

Obesity is a risk factor for HT (14). Song et al. found that obesity increases the risk of overt and subclinical hypothyroidism, thyroid peroxidase antibody (TPOAb) positivity, but not thyroglobulin antibody (TgAb) positivity (16). Yan et al. found that obesity with HT had a higher incidence of subclinical hypothyroidism and TgAb positivity than HT subjects (57). In addition, obese children and adolescents with autoimmune thyroid diseases showed higher thyroid-stimulating hormone (TSH), lower thyroid hormone levels, a higher risk of hypothyroidism, and no association with antibody levels (58). Maria et al. found that overweight or obese women with HT have higher oxidative stress levels compared with women with normal BMI with HT (59). All these research studies suggest that treating obesity may be a valuable therapy for treating HT (Figure 6).

Obesity is also related to efferocytosis. Li et al. found that efferocytosis was defective in obese mice (60). The mechanism of defective efferocytosis in obesity has also been widely studied. Reduced PPARG signaling has been reported as one of the factors contributing to defective efferocytosis (20). Luo et al. mentioned that PPARG expression levels were reduced in obese mice, which were further found to be associated with defective macrophage erythropoietin signaling (61). Suresh Babu et al. showed that efferocytosis was altered in macrophages due to the shedding of MERTK by ADAM17, which functions as the main protease, using obese and diabetic mice (62, 63). A clinical study on centripetal obesity further confirmed that obesity, particularly when fat is predominantly deposited around the abdominal area, is associated with the cleavage of MERTK by ADAM17 to produce soluble MERTK (64) (Figure 6).

Impaired efferocytosis has been implicated as a pathogenic mechanism in several ADs, and enhancing efferocytosis has emerged as a potential therapeutic strategy for these conditions (65–70). In systemic lupus erythematosus, defective efferocytosis is closely associated with aberrations in PPAR signaling, LXR signaling, ABCA1 expression, and C1q membrane protein deficiency (71, 72). Similarly, macrophages derived from non-obese diabetic (NOD) mice, an established animal model of type 1 diabetes, exhibit defective efferocytosis both *in vivo* and *in vitro* (70). In systemic sclerosis, efferocytosis is frequently impaired due to the presence of inhibitory IgG anti-apoptotic cell antibodies and phagocyte dysfunction (69). In osteoarthritis, impaired efferocytosis and elevated levels of apoptotic cells have been observed in synovial tissues, which have been linked to the deficiency of membrane-bound TAM receptors (Tyro3, AXL, and MERTK) (73). In recent years, efferocytosis-informed nanomimetics have demonstrated therapeutic efficacy in murine models of rheumatoid arthritis (74, 75). Regulating macrophage polarization is an effective means to treat inflammatory bowel disease (67), and efferocytosis will promote macrophage M2 polarization (68). In brief, efferocytosis plays a crucial role in several ADs. HT is also a form of AD and is likely to be similarly affected by efferocytosis dysfunction. Therefore, it is reasonable to infer that the promotion of efferocytosis may be one of the effective ways of treating HT (Figure 6).

Since this study relies solely on network pharmacology methods, it inevitably possesses certain limitations. The relationship between efferocytosis and obesity-associated HT has not been supported by experimental and clinical studies. Currently, there is a scarcity of studies on HT as a risk factor for obesity; it still needs further experimental and clinical research. The treatment of quercetin for HT also needs to be confirmed by further clinical studies.

## 5 Conclusion

The therapeutic effect of five frequently prescribed herbs in treating obesity-associated HT may be achieved through the regulation of efferocytosis. Most of the results from network pharmacology and molecular docking techniques have pointed to efferocytosis as a potential pathway to treat obesity-associated HT. Quercetin is one of the potential ingredients related to the efferocytosis pathway, and it binds well with most of the key efferocytosis-related proteins.

## Data Availability

The original contributions presented in the study are included in the article/Supplementary material, further inquiries can be directed to the corresponding author.

## Supplementary material

SUPPLEMENTARY TABLE S1 Information for potential targets of five herbs and obesity-associated HT.

SUPPLEMENTARY TABLE S2 Venn diagram data from five-herb GO enrichment analysis.

SUPPLEMENTARY TABLE S3 Venn diagram data from four-herb pair GO enrichment analysis.

SUPPLEMENTARY TABLE S4 Bubble figure and Venn diagram data from KEGG enrichment analysis.

SUPPLEMENTARY TABLE S5 Active ingredient analysis data.
